# Supplementary material for: Urodynamics Are Essential to Predict the Risk for Upper Urinary Tract Damage after Acute Spinal Cord Injury
Source: Biomedicines. 2023 Jun 17;11(6):1748. doi: 10.3390/biomedicines11061748 (PMC10296654; doi:10.3390/biomedicines11061748)
Supplement: Supplementary file 1 [file biomedicines-11-01748-s001.zip › biomedicines-2421101-supplementary.pdf]

Birkhäuser V, Anderson CE, Kozomara M, Bywater M, Gross O, Kiss S, Knüpfer SC, Koschorke M, Leitner L, Mehnert U, Sadri H, Sammer U, Stächele L, Tornic J, Liechti MD, Brinkhof MWG, Kessler TM. Urodynamics are essential to predict the risk for upper urinary tract damage after acute spinal cord injury; Biomedicines, 2023

Supplementary file

This supplementary file consists of three parts, the first of which contains detailed information on the three strategies that were used to address missing data, in particular loss-to-follow-up, including: a.) Multiple imputation (MI); b.) Inverse probability weights (IPWs); c.) "Informed" outcome assignment. The second part of the file contains the results from logistic regression models predicting maximum detrusor pressure during the storage phase (pDetmax) ≥40 cmH_2O within the first year after SCI, and model performance under different missing data assumptions. The third part contains results of the sensitivity analyses with the outcome of (pDetmax) ≥40 cmH_2O or use of antimuscarinics within the first year after SCI.

Supplement S1a. Multiple Imputation

Methodology

The approach to address missing data used in the primary analyses was to employ multiple imputation (MI) under the assumption of missing at random. There were 30/97 (31%) patients missing data in at least one of the variables employed in the prognostic model (Table S1). Of the 24 patients who did not return for the one-year follow-up, four had already developed the outcome, maximum detrusor pressure (pDetmax) ≥40 cmH_2O during the storage phase, so effectively 20 of these observations were missing data.

The MI approach was multiple imputation by chained equations, which was performed in Stata (version 16.1, College Station, TX, USA). Twenty imputed datasets were generated and the number of burn-in iterations was set to 200. The model contained lower extremity motor score (LEMS), Spinal Cord Independence Measure version III respiration and sphincter management subscale as well as total score, upper extremity motor score (UEMS), neurological level, American Spinal Injury Association Impairment Scale (AIS) grade, the highest light touch score from the S3 dermatome, and the outcome, pDetmax ≥40 cmH_2O during the storage phase, as well as age and sex.

Table S1. Missing data in variables selected for imputation

Variable	n (%) missing data
Outcome variable – status 12 months after SCI	
Storage pDetmax ≥40 cmH_2O	20 (21%)
Predictor variables – status 1 month after SCI	
Upper extremity motor score (UEMS)	5 (5)
Lower extremity motor score (LEMS)	6 (6)
Light touch score of the S3 dermatome (highest)	6 (6)
Neurological level	5 (5)
AIS grade	5 (5)
SCIM respiration and sphincter management	2 (2)

In the MI analysis, logistic regression models were run on each individual data set, using "Rubin's Rules" to combine estimates and standard errors across MI sets. The area under the receiver operator characteristic curve (aROC) was calculated by comparing the predictions from the combined coefficients to the actual data [25,26]. Similarly, Figure 1 (main text) displays aROC curves based on the MI estimate – plotting results of the MI-based predictions against the actual data. Internal validation of MI models was performed using a bootstrapping approach [23]. The bootstrap validation (1000 iterations) was performed after imputation on each of the 20 data sets, and the model performance statistics – optimism-corrected C-statistics, Brier score and expected to observed ratio –

were pooled using Rubin's rules [25]. Lasso regression to confirm the variable selection strategy was performed on each imputed data set, and the most commonly selected model is reported.

Supplement S1b. Inverse probability weighting (IPW)

Methodology

Inverse probability weighting was the second approach utilized to address the loss-to-follow-up in the full population of patients with an acute spinal cord injury (SCI) that underwent urodynamic investigation (UDI). A logistic regression model was generated, and return for the 12-month follow-up was coded as '1'. Covariates included demographic characteristic (categorized age, sex), and SCI characteristics (SCI etiology [traumatic spinal cord injury or ischemic spinal cord injury], American Spinal Injury Association Impairment Scale [AIS] grade [A, B/C, D], neurological level [categorized – cervical, thoracic, lumbar]), and year of SCI. Information on SCI characteristics was taken from the assessment that was performed approximately one month after SCI where possible, SCI information was taken from another time point, preferentially a later one, when one-month data were missing (n=5). There were no other missing data concerns in the predictor variables. The predictions produced by the regression model (propensity scores) were used to generate the inverse probability weights (IPWs).

Results

The study population included 97 persons 73 of whom (75%) returned for a 12-month follow-up visit. In adjusted analysis, older persons (age ≥76) were less likely to return for the 12-month follow-up visit. Full results from this logistic regression model have previously been reported in the supplemental material from Kozomara, Birkhäuser et al [15]. Figure S1 shows histograms of the propensity score distribution, stratified according to return for the 12-month follow-up visit. Inverse probability weight characteristics for the population who returned for the 12-month follow-up were: mean 1.28 (range: 1.01-2.20), the inverse mean (1/1.28) was 78%. The mean-standardized range of the IPWs was 0.79-1.73, and the standardized ratio, indicative of the largest proportional difference between two participants in the data set, was 2.19.

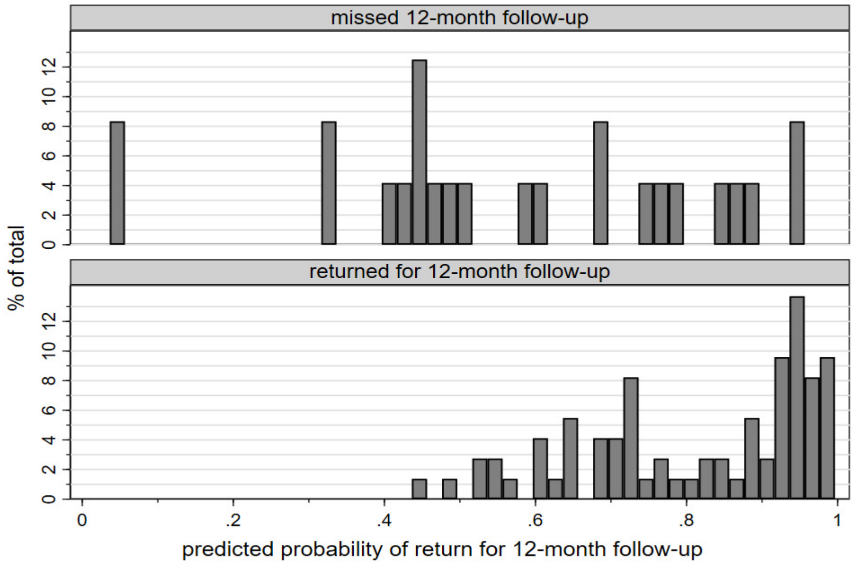

Figure S1. Histograms showing the distribution of propensity scores for persons who missed the 12-month follow-up and persons who returned for 12-month follow-up. Percent of total refers to the respective population.

Supplement S1c. Informed imputation for storage pDetmax ≥40 cmH_2O status

Methodology

The informed imputation was a sensitivity analysis designed to address concerns that the assumptions needed for IPW and MI analyses were not fulfilled in this data set. Table 1 of the main text provides evidence that loss-to-follow-up was associated with the outcome variable – storage pDetmax ≥40 cmH_2O was detected in 34/73 (47%) of the patients that returned for the 12-month follow-up, but only in 4/24 (17%) of the patients who did not. The graph of the propensity scores (Figure S1), is further indication that the coverage of some patients lost-to-UDI-follow-up could be inadequate with the IPWs. To address these issues, further information from the clinical records was utilized by an experienced neuro-urologist to assign patients to the most-likely outcome. In 10 cases the patient continued to return to the clinic but did not undergo a UDI follow-up, and 1 patient had a UDI with no detection of storage pDetmax ≥40 cmH_2O more than 2 years after SCI. In 3 cases that transferred to other urology clinics reports from the other clinics were available, and UDI findings were mentioned in 2/3 cases. Four patients were completely lost-to-follow-up, although in two of those cases the probability of pDetmax ≥40 cmH_2O was considered to be low based on information from UDI from earlier time points (e.g. incontinence starting at detrusor pressures well below 40 cmH_2O).

The 20 patients who had not developed storage pDetmax ≥40 cmH_2O before the time of loss-to-follow-up were the focus of this investigation. Two patients were excluded because the reason for loss-to-follow-up was death. Of the 18 remaining patients, using additional information from the clinical record, it was possible to come to a reasonable assignment for 13/18. Notably, 12 of these patients were thought to be at very low risk of developing storage pDetmax ≥40 cmH_2O. For the sensitivity analyses the complete case logistic regression models were run twice and the five patients where there was uncertainty were all assigned to the same group - first storage pDetmax ≥40 cmH_2O absent, then present. The findings from the two sensitivity analyses were very similar, the results of the first are presented in the main body of the text.

References

15. Kozomara, M.; Birkhäuser, V.; Anderson, C.E.; Bywater, M.; Gross, O.; Kiss, S.; Knüpfer, S.C.; Koschorke, M.; Leitner, L.; Mehnert, U.; et al. Neurogenic Lower Urinary Tract Dysfunction in the First Year After Spinal Cord Injury: A Descriptive Study of Urodynamic Findings. *J. Urol.* **2023**, *209*, 225–232.
23. Fernandez-Felix, B.; García-Esquinas, E.; Muriel, A.; Royuela, A.; Zamora, J. Bootstrap internal validation command for predictive logistic regression models. *Stata J.* **2021**, *21*, 498–509.
25. Sterne, J.A.; White, I.R.; Carlin, J.B.; Spratt, M.; Royston, P.; Kenward, M.G.; Wood, A.M.; Carpenter, J.R. Multiple imputation for missing data in epidemiological and clinical research: Potential and pitfalls. *BMJ* **2009**, *338*, b2393.
26. Spratt, M.; Carpenter, J.; Sterne, J.A.; Carlin, J.B.; Heron, J.; Henderson, J.; Tilling, K. Strategies for multiple imputation in longitudinal studies. *Am. J. Epidemiol.* **2010**, *172*, 478–487.

Table S2. Model performance – area under the receiver operating characteristic curve (aROC), using four different strategies to account for loss-to-follow-up. All models were tested with all participants and then excluding persons who had already developed maximum detrusor pressure (pDetmax) ≥ 40 cmH_2O during the storage phase 1 month after spinal cord injury (SCI). In addition to the multiple imputation (MI) approach (n=97 all, n=76 no outcome one month after SCI), results from a complete case model (n=68 all, n=50 no outcome at one month after SCI), an inverse probability weight (IPW) model that used the same population, and informed outcome assignment (n=81 all, n=63 no outcome 1 month after SCI) are also displayed.

Model	All patients, aROC	No outcome at 1 month after SCI, aROC
Model 1		
MI	0.533 (0.402 - 0.663)	0.578 (0.405 - 0.750)
Complete Case	0.514 (0.372 - 0.655)	0.576 (0.382 - 0.769)
IPW	0.486 (0.345 - 0.628)	0.576 (0.382 - 0.769)
Informed	0.537 (0.402 - 0.672)	0.601 (0.413 - 0.789)
Model 2		
MI	0.649 (0.523 - 0.775)	0.690 (0.528 - 0.852)
Complete Case	0.654 (0.521 - 0.788)	0.658 (0.473 - 0.843)
IPW	0.645 (0.510 - 0.780)	0.652 (0.468 - 0.836)
Informed	0.630 (0.502 - 0.758)	0.643 (0.467 - 0.819)
Model 3		
MI	0.790 (0.689 - 0.890)	0.732 (0.601 - 0.864)
Complete Case	0.769 (0.659 - 0.878)	0.677 (0.529 - 0.824)
IPW	0.782 (0.674 - 0.889)	0.677 (0.529 - 0.824)
Informed	0.768 (0.666 - 0.870)	0.678 (0.539 - 0.816)
Model 4		
MI	0.782 (0.676 - 0.888)	0.731 (0.596 - 0.866)
Complete Case	0.790 (0.682 - 0.899)	0.763 (0.622 - 0.904)
IPW	0.793 (0.685 - 0.901)	0.755 (0.613 - 0.896)
Informed	0.793 (0.695 - 0.891)	0.749 (0.608 - 0.890)

Model 1: lower extremity motor score (LEMS); Model 2: LEMS, highest light touch score of the S3 dermatome, Spinal Cord Independence Measure (SCIM) III respiratory-sphincter subscale; Model 3: upper extremity motor score (UEMS) and sex; Model 4: neurological level, American Spinal Injury Association Impairment Scale (AIS) grade, sex
pDetmax=maximum detrusor pressure (storage phase), SCI=spinal cord injury, UDI=urodynamic investigation

Table S3. Predictors of maximum detrusor pressure during the storage phase (pDetmax) ≥40 cmH₂O during the first year after SCI. Logistic regression results from four different candidate prediction models. pDetmax ≥40 cmH₂O during the first year after SCI is coded as '1'. Results from multiple imputation models (primary analysis), complete case models, and inverse probability weighting (IPW) to account for loss-to-follow-up are shown. The analysis was performed in two separate populations – first all participants, and then restricting the analysis to persons who did not have a pDetmax ≥40 cmH₂O at baseline.

	**All participants**						**Excluding participants with pDetmax ≥40 1 month after SCI**					
	**Multiple Imputation**		**Complete Case**		**Using IPW**		**Multiple Imputation**		**Complete Case**		**Using IPW**	
**Predictor**	**Adjusted Odds ratio (aOR)**	**p-value**	**Adjusted Odds ratio (aOR)**	**p-value**	**Adjusted Odds ratio (aOR)**	**p-value**	**Adjusted Odds ratio (aOR)**	**p-value**	**Adjusted Odds ratio (aOR)**	**p-value**	**Adjusted Odds ratio (aOR)**	**p-value**
**Model 1**												
LEMS	1.00 (0.98 - 1.02)	0.67	1.00 (0.97 - 1.02)	0.75	1.01 (0.98 - 1.04)	0.55	0.99 (0.97 - 1.02)	0.678	0.99 (0.95 - 1.02)	0.38	0.98 (0.95 - 1.01)	0.22
**Model 2**												
LEMS	1.00 (0.98 - 1.03)	0.76	1.00 (0.96 - 1.03)	0.87	1.01 (0.97 - 1.05)	0.72	1.01 (0.98 - 1.05)	0.43	0.98 (0.94 - 1.03)	0.53	0.98 (0.93 - 1.03)	0.38
S3 light touch score		0.32		0.20		0.041		0.47		0.55		0.41
Absent	0.17 (0.03 - 0.92)		0.18 (0.02 - 1.31)		0.04 (0.00 - 0.50)		0.09 (0.01 - 0.84)		0.23 (0.02 - 3.34)		0.13 (0.01 - 2.61)	
Altered	0.19 (0.05 - 0.77)		0.25 (0.05 - 1.25)		0.08 (0.01 - 0.68)		0.10 (0.01 - 0.71)		0.30 (0.03 - 3.08)		0.21 (0.02 - 2.75)	
Normal	REF		REF		REF		REF		REF		REF	
SCIM respiratory-sphincter subscale	0.93 (0.87 - 0.98)	0.012	0.95 (0.89 - 1.03)	0.22	0.90 (0.81 - 1.00)	0.058	0.88 (0.81 - 0.96)	<0.01	0.97 (0.87 - 1.08)	0.22	0.96 (0.85 - 1.08)	0.46
**Model 3**												
Sex		<0.001		<0.01		<0.001		<0.01		0.035		0.030
Female	REF		REF		REF		REF		REF		REF	
Male	7.61 (2.62 - 22.06)		8.81 (2.30 - 33.78)		12.82 (2.98 - 55.23)		4.86 (1.52 - 15.51)		5.74 (1.13 - 29.23)		5.46 (1.18 - 25.20)	
UEMS	0.95 (0.91 - 0.99)	0.010	0.95 (0.89 - 1.02)	0.17	0.92 (0.84 - 1.00)	0.048	0.95 (0.92 - 0.99)	0.028	0.98 (0.90 - 1.07)	0.65	0.97 (0.88 - 1.06)	0.49
**Model 4**												
Sex		<0.0001		<0.001		<0.001		<0.01		0.028		0.031
Female	REF		REF		REF		REF		REF		REF	
Male	10.14 (3.23 - 31.83)		12.55 (3.02 - 52.19)		18.96 (3.90 - 92.23)		6.37 (1.87 - 21.68)		6.84 (1.23 - 38.18)		6.69 (1.19 - 37.68)	
Neurological level		0.41		0.30		0.10		0.83		0.85		0.72
Cervical (C1-C8)	6.41 (1.36 - 30.32)		3.93 (0.70 - 22.00)		7.08 (1.17 - 42.76)		3.33 (0.63 - 17.62)		1.43 (0.17 - 11.86)		1.73 (0.20 - 15.25)	
Thoracic (T1-T12)	3.35 (0.66 - 17.00)		2.82 (0.47 - 17.05)		3.69 (0.54 - 25.17)		2.34 (0.43 - 12.77)		1.72 (0.26 - 11.14)		2.20 (0.32 - 15.05)	
Lumbar (L1-L5)	REF		REF		REF		REF		REF		REF	
AIS Grade		0.42		0.16		0.054		0.644		0.31		0.15
A	0.42 (0.12 - 1.42)		0.40 (0.09 - 1.73)		0.28 (0.05 - 1.47)		0.45 (0.11 - 1.81)		0.66 (0.09 - 4.72)		0.83 (0.11 - 6.07)	
B/C	2.13 (0.56 - 8.16)		2.34 (0.44 - 12.62)		2.90 (0.50 - 16.94)		2.01 (0.47 - 8.61)		3.25 (0.44 - 24.31)		5.68 (0.72 - 44.47)	
D	REF		REF		REF		REF		REF		REF	

AIS=American Spinal Injury Association (ASIA) Impairment Scale, C=cervical, L=lumbar, LEMS=lower extremity motor score, S=sacral, SCIM=Spinal Cord Independence Measure III, T=thoracic, UEMS=upper extremity motor score

Table S4. Sensitivity analysis: Predictors of maximum detrusor pressure during the storage phase (pDetmax) ≥40 cmH₂O or antimuscarinic use during the first year after SCI. Logistic regression results from four different candidate prediction models. pDetmax ≥40 cmH_2O during the first year after SCI or antimuscarinic use is coded as '1'. Complete case (CC) models, as well as those using inverse probability weighting (IPW) to account for loss-to-follow-up are shown. The analysis was performed in two separate populations –all participants and then restricting the analysis to persons who did not have a pDetmax ≥40 cmH_2O or start antimuscarinics at the baseline time point, 1 month after SCI.

	**All participants**				**Excluding participants with pDetmax ≥40 or antimuscarinic use 1 month after SCI**			
	**Complete Case**		**Using IPW**		**Complete Case**		**Using IPW**	
**Predictor**	**Adjusted Odds ratio (aOR)**	**p-value**	**Adjusted Odds ratio (aOR)**	**p-value**	**Adjusted Odds ratio (aOR)**	**p-value**	**Adjusted Odds ratio (aOR)**	**p-value**
**Model 1**								
LEMS	0.97 (0.95 - 1.00)	0.067	0.98 (0.95 - 1.02)	0.37	0.97 (0.94 - 1.01)	0.16	0.97 (0.93 - 1.01)	0.11
**Model 2**								
LEMS	1.00 (0.96 - 1.05)	0.94	1.01 (0.96 - 1.07)	0.72	1.00 (0.94 - 1.05)	0.88	0.99 (0.94 - 1.05)	0.71
S3 light touch score		0.22		0.079		0.28		0.25
Absent	4.14 (0.30 - 56.39)		1.51 (0.08 - 27.86)		6.41 (0.38 - 107.39)		4.70 (0.26 - 86.27)	
Altered	0.65 (0.14 - 3.00)		0.23 (0.04 - 1.42)		1.00 (0.16 - 6.04)		0.67 (0.10 - 4.36)	
Normal	REF		REF		REF		REF	
SCIM respiratory-sphincter subscale	0.96 (0.89 - 1.03)	0.23	0.92 (0.84 - 1.00)	0.059	0.99 (0.90 - 1.07)	0.23	0.98 (0.89 - 1.07)	0.60
**Model 3**								
Sex		<0.01		<0.01		0.038		0.044
Female	REF		REF		REF		REF	
Male	5.65 (1.63 - 19.55)		6.40 (1.78 - 22.98)		4.51 (1.09 - 18.72)		4.55 (1.04 - 19.87)	
UEMS	0.93 (0.88 - 0.98)	<0.01	0.91 (0.85 - 0.98)	0.013	0.92 (0.86 - 0.99)	0.027	0.93 (0.87 - 1.00)	0.061
**Model 4**								
Sex		0.013		<0.01		0.24		0.12
Female	REF		REF		REF		REF	
Male	5.32 (1.42 - 19.96)		9.45 (2.21 - 40.39)		2.60 (0.53 - 12.67)		3.47 (0.72 - 16.73)	
Neurological level		0.95		0.86		0.69		0.84
Cervical (C1-C8)	1.10 (0.13 - 9.47)		1.60 (0.16 - 15.83)		0.51 (0.06 - 4.03)		0.54 (0.07 - 4.17)	
Thoracic (T1-T12)	1.32 (0.16 - 11.10)		1.90 (0.19 - 18.65)		0.37 (0.04 - 3.60)		0.61 (0.06 - 6.04)	
Lumbar (L1-L5)	REF		REF		REF		REF	
AIS Grade		0.17		0.23		0.14		0.10
A	5.87 (0.66 - 52.18)		5.73 (0.49 - 66.48)		8.98 (0.86 - 94.14)		10.24 (0.88 - 118.64)	
B/C	3.06 (0.55 - 16.92)		3.21 (0.51 - 20.12)		2.79 (0.40 - 19.68)		3.98 (0.54 - 29.12)	
D	REF		REF		REF		REF	

AIS=American Spinal Injury Association (ASIA) Impairment Scale, C=cervical, L=lumbar, LEMS=lower extremity motor score, S=sacral, SCIM=Spinal Cord Independence Measure III, T=thoracic, UEMS=upper extremity motor score

Table S5. Sensitivity analysis: Model testing – area under the receiver operating characteristic curve (aROC) for maximum detrusor pressure during the storage phase (pDetmax) ≥40 cmH_2O or antimuscarinic use. All models were tested with all participants and then excluding persons who had already developed maximum detrusor pressure (pDetmax) ≥ 40 cmH_2O during the storage phase or started antimuscarinic medication 1 month after spinal cord injury (SCI). Results are from a complete case model (n=68 all, n=40 no outcome at one month after SCI), and an inverse probability weight (IPW) model that used the same population.

Model	All patients, aROC	No outcome at 1 month after SCI, aROC
Model 1		
Complete Case	0.636 (0.494 - 0.777)	0.624 (0.447 - 0.801)
IPW	0.636 (0.494 - 0.777)	0.541 (0.292 - 0.791)
Model 2		
Complete Case	0.711 (0.568 - 0.855)	0.705 (0.540 - 0.869)
IPW	0.730 (0.595 - 0.864)	0.697 (0.530 - 0.864)
Model 3		
Complete Case	0.746 (0.620 - 0.873)	0.705 (0.544 - 0.865)
IPW	0.749 (0.623 - 0.874)	0.666 (0.508 - 0.824)
Model 4		
Complete Case	0.803 (0.676 - 0.930)	0.780 (0.635 - 0.925)
IPW	0.796 (0.668 - 0.925)	0.777 (0.627 - 0.928)
